# The incidence of coronary anomalies on routine coronary computed tomography scans

**DOI:** 10.5830/CVJA-2013-066

**Published:** 2013-11

**Authors:** Kanber Ocal Karabay, Abdulmelik Yildiz, Bayram Bagirtan, Gurkan Geceer, Ender Uysal

**Affiliations:** Department of Cardiology, Kadikoy Florence Nightingale Hospital, Istanbul, Turkey; Department of Cardiology, Avrupa Safak Hospital, Istanbul, Turkey; Department of Cardiology, Avrupa Safak Hospital, Istanbul, Turkey; Department of Radiology, Kadikoy Florence Nightingale Hospital, Istanbul, Turkey; Department of Radiology, Sisli Etfal Research and Training Hospital, Istanbul, Turkey

**Keywords:** coronary artery anomaly, coronary CT angiography, coronary artery fistula, coronary aneurysm, myocardial bridging

## Abstract

**Objective:**

This study aimed to assess the incidence of coronary anomalies using 64-multi-slice coronary computed tomography (MSCT).

**Methods:**

The diagnostic MSCT scans of 745 consecutive patients were reviewed.

**Results:**

The incidence of coronary anomalies was 4.96%. The detected coronary anomalies included the conus artery originating separately from the right coronary sinus (RCS) (*n* = 8, 1.07%), absence of the left main artery (*n* = 7, 0.93%), a superior right coronary artery (RCA) (*n* = 7, 0.93%), the circumflex artery (CFX) arising from the RCS (*n* = 4, 0.53%), the CFX originating from the RCA (*n* = 2, 0.26%), a posterior RCA (*n* = 1, 0.13%), a coronary fistula from the left anterior descending artery and RCA to the pulmonary artery (*n* = 1, 0.13%), and a coronary aneurysm (*n* = 1, 0.13%).

**Conclusions:**

This study indicated that MSCT can be used to detect common coronary anomalies, and shows it has the potential to aid cardiologists and cardiac surgeons by revealing the origin and course of the coronary vessels.

## Abstract

The incidence of coronary anomalies (CCAs) in a typical angiographic study was 1.3%.^1^ Studies have been conducted on CCAs using conventional invasive coronary angiography in highly selected groups of patients but these studies may not reflect the true incidence of CCAs.

Although the majority of CCAs are benign and incidentally detected during conventional angiography, certain CCAs may cause syncope, heart failure or sudden death, especially among young athletes.^2^,^3^ The US National Registry of Sudden Death in Athletes at the Minneapolis Heart Institute Registry found that CCAs were the second most common cause of sudden cardiac death (out of 17% of the population who died of cardiac-related causes).^4^

Although conventional invasive coronary angiography is considered the gold standard for the diagnosis of CCAs, transthoracic two-dimensional echocardiography, transoesophageal echocardiography, magnetic resonance imaging and multi-slice computed tomography (MSCT) can all identify for diagnosis, CCAs in certain groups of patients.5-10 Transthoracic twodimensional echocardiography may depict the origin of the coronary arteries, especially the left main artery, but successful detection of coronary anomalies depends on the age and size of the patient.^5^,^6^

Transoesophageal echocardiography has an increased success rate of identifying coronary anomalies in comparison with two-dimensional echocardiography. Nevertheless, the position of the transducer, cardiac motion, and the curvilinear course of the vessel all affect visualisation of coronary anomalies. Moreover, transoesophageal echocardiography is a semi-invasive method and is time consuming.^6^,^7^

Magnetic resonance (MR) imaging provides an accurate assessment of the course of anomalous coronary arteries.^8^,^9^ However, this technique cannot be performed in patients with pacemakers, certain types of arrhythmias or defibrillating devices, and it may be difficult to perform in claustrophobic patients. Furthermore, the spatial resolution of MR imaging is substantially inferior to that of the newest generation of CT scanners.^10^

Myocardial bridging (MB) is defined as the compression of a coronary artery during systole while it is normal in diastole. MB has been linked to serious cardiac events.^11^ The incidence of myocardial bridging in the population varies substantially according to invasive coronary angiography (13%) and autopsy (15–85%).^12^,^13^ The reported incidence of MB has increased up to 44% when using 64-MSCT.^14^ Because of its ability to cause serious cardiac events, diagnosing MB is clinically important.

MSCT is a minimally invasive method that provides excellent temporal and spatial resolution of the coronary arteries. There have been a limited number of studies evaluating CCAs and MB with 64-MSCT. The aim of this study was to assess the incidence of CCAs and MB using 64-MSCT in a relatively large population.

## Methods

Our institutional ethics committee approved the study protocol. Two experienced radiologists and three invasive cardiologists who were familiar with CCA retrospectively interpreted 745 diagnostic scans of 745 consecutive patients taken between July 2007 and December 2011 at Istanbul Kadikoy Florence Nightingale Hospital. Previous reports were not used.

Exclusion criteria were severe arrhythmia, previous serious allergic reaction to the contrast medium, pregnancy, and renal and respiratory failure. None of the patients had repeat CT scans. The indications for MSCT scans included the presence of chest pain, abnormal stress testing, the presence of ischaemia on non-invasive tests, coronary artery disease screenings and the determination of patency of bypass grafts or stents.

Patients received a 50- to 100-mg oral dose of metoprolol one hour prior to the scan, with additional intravenous (IV) metoprolol administered immediately prior to the scan if necessary. The target heart rate (HR) was less than 70 beats per minute, under close cardiac monitoring for heart rate and blood pressure. If necessary, oral alprazolam (0.5–1 mg) was administered 60 min prior to the procedure. All patients received sublingual isosorbide dinitrate immediately prior to starting the scan protocol.

Computed tomography coronary angiography was carried out using a 64-MSCT scanner (General Electric Light Speed VCT scanner, Waukesha, WI, USA) after IV injection of 80 ml of non-ionic contrast medium (Iopamiro 370; Bracco, Milan, Italy) as a bolus dose at a rate of 6 ml/s with retrospective ECG gating. The scan field was extended from the proximal aorta to the cardiac apex.

The imaging parameters were as follows: detector collimation of 64 × 0.625 mm, tube voltage 120 kV, current 500–800 mA, gantry rotation time 350 ms, pitch 0.20, and slice thickness 0.625 mm. Retrospective ECG-gated images were obtained during one held breath. These images were evaluated with multiplanar reconstruction, maximum-intensity projection and three-dimensional volume-rendering methods. The 75% phase during diastole was found to be optimal for the analysis of anomalies of the left coronary arteries. The right coronary artery (RCA) was evaluated at either the 45 or 75% phase of the cardiac cycle, depending on which phase presented the least amount of motion.

## Results

Of the 745 patients, 276 were female and 449 were male. The mean age was 54.9 ± 11.3 years. The indications for MSCT scans included the presence of chest pain (*n* = 250, 33.5%), or ischaemia on non-invasive tests (ECG, treadmill stress test or myocardial perfusion scintigraphy) (*n* = 153, 20.5%), coronary artery disease screenings (*n* = 220, 29.5%), and the determination of patency of bypass grafts (*n* = 76, 10.2%) or stents (*n* = 46, 6.1%). The incidence of diabetes, hypertension, hyperlipidaemia, family history and smoking were 14.7, 34.4, 34.7, 9.3 and 23.2%, respectively. The patients’ characteristics are shown in Table 1.

**Table 1:** Patients’ Characteristics*

*Characteristics*	*Number*	*%*
Female	276	37
Male	449	60.2
Hypertension	257	34.4
Hyperlipidaemia	258	34.6
Diabetes	110	14.7
Smoking	173	23.2
Prior stenting	46	6.1
Prior bypass	76	10.2

*Median age ± SS = 54.9 ± 11.3 years.

Six patients with CCAs, and 16 with MB had atypical chest pain and dyspnoea. None of the patients with CCAs or MB suffered from syncope.

Right dominance was observed in 563 patients (75.5%), balanced dominance in 110 (14.7%), and left dominance in 72 patients (9.6%). We identified 176 patients who had evidence of either a coronary anomaly (4.42%, *n* = 33) or myocardial bridging (19.1%, *n* = 143) (Table 2). The majority of patients with coronary anomalies were male (63.63%, *n* = 21).

**Table 2:** Coronary Anomalies

	*Number*	*Incidence (%)*	*Anomalies (%)*
Benign anomalies			
CA from RCS	8	1.07	24.24
Absence of LMA	7	0.93	21.21
Posterior RCA	1	0.13	3.03
Potentially clinically significant anomalies			
Superior RCA	7	0.93	21.21
CFX from RCS	4	0.53	12.12
CFX from RCA	2	0.26	6.06
Coronary fistula	1	0.13	3.03
Coronary aneurysm	1	0.13	3.03

CA: conus artery; RCS: right coronary sinus; LM: left main artery; RCA: right coronary artery.

The patients with detected coronary anomalies included eight with the conus artery originating separately from the right coronary sinus (RCS) (1.07%), seven with absence of the left main artery (0.93%), seven with a superior right coronary artery (RCA) (0.93%), four with the circumflex artery (CFX) arising from the RCS (0.53%), two with the CFX originating from the RCA (0.26%), one with a posterior origin of the RCA (0.13%), one with a coronary fistula from the left anterior descending artery and RCA to the pulmonary artery (0.13%), and one with a coronary artery aneurysm (0.13%). Atherosclerosis was observed in four patients with an anomalous CFX arising from the RCA and five with a superior RCA. The other patients with coronary anomalies did not have any atherosclerosis.

Of the six patients with anomalous origins of the CFX from either the RCA or RCS, three patients had mild atherosclerosis in all the coronary arteries including the anomalous CFX, one had atherosclerosis in only the anomalous CFX, and one had atherosclerosis in the other vessels but not in the anomalous CFX. One patient with an anomalous CFX did not have any atherosclerosis in any coronary artery. Four patients with a superior RCA had atherosclerosis in the anomalous and normal vessels, and one had atherosclerosis in only the superior RCA.

One patient with a superior RCA origin had atherosclerosis in the normal originating coronary arteries but not in the anomalous RCA. One patient had no atherosclerosis in any of the vessels. One patient with a superior origin had a history of stenting, both in the superior RCA and the left anterior descending (LAD) artery. One patient with a posterior RCA had no atherosclerosis in the anomalous or normal vessels or the coronary arteries.

Myocardial bridging was mostly observed in the LAD (93.7%, *n* = 134), intermediate artery (1.39%, *n* = 2), obtuse margin artery (1.39%, *n* = 2), first diagonal artery (0.6%, *n* = 1), second diagonal artery (0.6%, *n* = 1) and RCA (0.6%, *n* = 1). In total, myocardial bridging was observed in 19.1% of the patients (*n* = 143) (Table 3).

**Table 3:** Myocardial Bridging

	*Number*	*Incidence (%)*	*Anomalies (%)*
IM	2	0.26	1.39
OM1	2	0.26	1.39
OM2	1	0.13	0.69
D1	1	0.13	0.69
D2	1	0.13	0.69
RCA	1	0.13	0.69

LAD: left anterior descending artery; IM: intermediary artery; OM1: first obtuse margin artery; OM2: second obtuse margin artery; D1: first diagonal artery; D2: second diagonal artery; RCA: right coronary artery sinus; MB: myocardial bridging.

## Discussion

In this study, the CCAs identified were mostly benign and mostly found in asymptomatic patients. Coronary anomalies are the second most common cause of sudden death in young adults.^4^ Determination of the incidence and presentation of high-risk CCAs is important. Most of our knowledge on the incidence and presentation of CCAs comes from a highly selected and biased population of patients undergoing invasive coronary angiography.

Although conventional coronary angiography is the gold standard for diagnosing CCA, its invasive nature, the difficulty in selective intubation of anomalous coronary arteries, and understanding the complex nature of the anomalous vessels during the procedure^15^-^17^ prevent its common usage to diagnose CCAs, especially in asymptomatic patients. Therefore conventional angiography may not be a true reflection of CCA incidence and presentation. MSCT, on the other hand, is minimally invasive, provides excellent temporal and spatial resolution, and has therefore recently become increasingly attractive for the diagnosis of CCAs.

MSCT studies, including our study, have reported higher incidence rates compared with conventional invasive angiography studies (1.3%) (Table 4). This may be related to increased diagnosis of CCA with MSCT compared with conventional angiography,^18^,^19^ or it may be due to the study groups used. For example, when compared with conventional angiography, MSCT studies include more asymptomatic patients. A study by Cademartiri *et al.* included 49.7% asymptomatic patients.^19^ In a study by Girzades *et al.*, 21.4% of patients with CCA and MB were asymptomatic.^20^ Similarly, our study included more asymptomatic (69.92%) than symptomatic patients. Recruiting asymptomatic patients in MSCT studies could be responsible for the apparent increased incidence of CCA.

**Table 4:** Studies Evaluating Coronary Anomalies Using 64-MSCT

	*No. patients*	*Incidence (%)*	*Absence LMA (%)*	*CFX from RCS/RCA (%)*	*MB (%)*	*Fistula (%)*	*Aneurysm (%)*	*Conus from aorta (%)*	*Right dominancy (%)*	*Superior RCA (%)*
Cademartiri *et al.*^19^	543	18.4	3.3	0.55	10.9	0.5	1.6	11.6	86.6	NA
Kosar *et al.*^24^	700	3.9	0.4	0.1	37	NA	NA	22	76	0.1
Girzadas *et al.*^20^	446	1.8	0.7	0.4	6.9	NA	0.4	NA	NA	NA
Andreini *et al.*^21^	2757	13.8	1.3	0.54	9.8	0.25	1.1	10.6	85	0.29
Erol *et al.*^25^	2096	1.96	0.43	0.43	3.18	0.33	0.74	NA	NA	0.38
Karabay *et al.* (this study)	745	4.96	0.93	0.79	19.1	0.13	0.13	1.07	75.5	0.93

LM: left main artery; CFX: circumflex artery; RCA: right coronary artery; RCS: right coronary sinus; MB: myocardial bridging.

In previous studies, right dominance has been the most common dominance. Similarly, the results of our study indicated right dominance in the majority of our patients (75.5%), but balanced dominance (14.1%) in our study was slightly higher than reported in previous studies (4.2–7%).^19^,^21^ Lack of consensus on the definition of a co-dominant coronary artery system may explain this discrepancy. We defined co-dominance as origination of the posterior descending artery from the RCA and the posterior left descending branch from the CFX, which is similar to most other studies.^22^ However, some authors describe co-dominance with regard to the artery supplying the inferolateral portion of the posterior septum. These authors state that if both arteries supply this portion of the septum, the system should be referred to as co-dominant.^23^

In our patient group, the detected CCAs accepted as benign, as in previous studies,^19^-^21^,^24^,^25^ were as follows: the conus artery separately arising from the RCS, an absence of the left main, superior RCA, posterior RCA and CFX, arising either from the RCS or RCA.^26^ It is important to diagnose a CFX originating from the RCS or RCA before valve surgery so as to avoid damage during cardiac surgery.^27^ In addition, although a superior RCA has generally been accepted as a benign anomaly, some cardiac events caused by this anomaly have previously been reported.^28^

A coronary artery aneurysm is a localised dilatation of the coronary artery exceeding the diameter of the adjacent normal segment by 50%,^29^ and is generally caused by atherosclerosis. With MSCT, Adreini *et al.* found 30 aneurysms among 2 757 patients.^21^ Individual aneurysms were observed in 19 of the 30 aneurysms, and the other 11 aneurysms were in the same patient. In our study, we found one aneurysm in the right ventricular branch of the right coronary artery, most likely caused by atherosclerosis, which was found in all the coronary arteries. The size of the aneurysm was small so medical therapy was chosen.

Erol *et al.* reported seven cases with fistulae (0.33% incidence).^25^ The fistulae were between the coronary and pulmonary artery (four cases), between the sino-atrial node artery and the left atrium (two cases) and between the right ventricular branch artery and the left ventricle (one case). When the Qp/Qs ratio is ≥ 2.0, surgical correction should be considered for coronary artery fistulae.^30^ Our patient had severe atherosclerosis in all the coronary vessels, which appeared to be the cause of the fistula. In this patient, the feeder vessels originated from both the LAD and RCA, and the ratio of total pulmonary blood flow to total systemic blood flow (Qp/Qs) was approximately 1. Therefore, medical therapy was chosen.

Myocardial bridging is when a segment of a coronary artery is covered by a bridge of myocardium. The reported incidence is between 28.7 and 58%.^31^,^32^ In our study, the incidence of MB was 19.1% and was mostly observed in the LAD artery (93.7%, *n* = 134). In most patients, myocardial bridging has an excellent survival rate of 97% at five years.^33^ Nevertheless, there have been reported associations with adverse cardiac events.^34^ None of the patients with MB had a history of cardiac events, and only 11.18% of our patients had symptoms of chest pain and dyspnoea during exercise. Beta-blocker therapy had been administered to those patients.

There were a few limitations to this study. It was a retrospective study that included only a single centre. The number of patients with CCAs was low in comparison with conventional angiography studies. The study population included patients who required a cardiac CT scan, therefore the incidence of coronary anomalies among the general population has not been answered by this study.

## Conclusions

Coronary anomalies and myocardial bridging were found to be common among patients undergoing cardiac CT scans at our institution, with an incidence of 23.6%. Most patients with CCA and MB who underwent MSCT were asymptomatic, and 77% of the patients with either CCA or MB were symptomatic.
